# Synthesis from hyperproperties

**DOI:** 10.1007/s00236-019-00358-2

**Published:** 2019-12-07

**Authors:** Bernd Finkbeiner, Christopher Hahn, Philip Lukert, Marvin Stenger, Leander Tentrup

**Affiliations:** Reactive Systems Group, Saarland University, Saarbrücken, Germany

## Abstract

We study the reactive synthesis problem for hyperproperties given as formulas of the temporal logic HyperLTL. Hyperproperties generalize trace properties, i.e., sets of traces, to *sets of sets* of traces. Typical examples are information-flow policies like noninterference, which stipulate that no sensitive data must leak into the public domain. Such properties cannot be expressed in standard linear or branching-time temporal logics like LTL, CTL, or $\hbox {CTL}^*$. Furthermore, HyperLTL subsumes many classical extensions of the LTL realizability problem, including realizability under incomplete information, distributed synthesis, and fault-tolerant synthesis. We show that, while the synthesis problem is undecidable for full HyperLTL, it remains decidable for the $\exists ^*$, $\exists ^*\forall ^1$, and the ${{ linear }}\;\forall ^*$ fragments. Beyond these fragments, the synthesis problem immediately becomes undecidable. For universal HyperLTL, we present a semi-decision procedure that constructs implementations and counterexamples up to a given bound. We report encouraging experimental results obtained with a prototype implementation on example specifications with hyperproperties like symmetric responses, secrecy, and information flow.

## Introduction

*Hyperproperties* [9] generalize trace properties in that they not only check the correctness of *individual* computation traces in isolation, but relate *multiple* computation traces to each other. $\text {HyperLTL}$ [8] is a logic for expressing temporal hyperproperties, by extending linear-time temporal logic (LTL) with *explicit* quantification over traces. $\text {HyperLTL}$ has been used to specify a variety of information-flow and security properties. Examples include classical security properties like non-interference [32] and observational determinism [38], as well as quantitative information-flow properties [7], symmetries in hardware designs, and formally verified error correcting codes [26]. For example, observational determinism can be expressed as the $\text {HyperLTL}$ formula  stating that, for every pair of traces, if the observable inputs are the same, then the observable outputs must be same as well.

In reactive synthesis, we automatically construct an implementation that is guaranteed to satisfy a given specification. A fundamental difference to verification is that there is no human programmer involved: in verification, the programmer would first produce an implementation, which is then verified against the specification. In synthesis, the implementation is directly constructed from the specification. Because there is no programmer, it is crucial that the specification contains *all* desired properties of the implementation: the synthesized implementation is guaranteed to satisfy the given specification, but nothing is guaranteed beyond that. The added expressive power of HyperLTL over LTL is very attractive for synthesis: with synthesis from hyperproperties, we can guarantee that the implementation does not only accomplish the desired functionality, but is also free of information leaks, is symmetric, is fault-tolerant with respect to transmission errors, etc.

More formally, the reactive synthesis problem asks for a *strategy*, that is a tree which branches on environment inputs and whose nodes are labeled by the system output. Collecting the inputs and outputs along a branch of the tree, we obtain a trace. If the set of traces collected from the branches of the strategy tree satisfies the specification, we say that the strategy *realizes* the specification. The specification is *realizable* iff there exists a strategy tree that realizes the specification. With LTL specifications, we get trees where the trace on each individual branch satisfies the LTL formula. With HyperLTL, we get trees where the traces between different branches are in a specified relationship. This is dramatically more powerful.

Consider, for example, the well-studied *distributed* version of the reactive synthesis problem, where the system is split into a set of processes, where each only sees a subset of the inputs. The distributed synthesis problem for LTL can be expressed as the standard (non-distributed) synthesis problem for HyperLTL, by adding for each process the requirement that the process output is *observationally deterministic* in the process input. HyperLTL synthesis thus subsumes distributed synthesis. The information-flow requirements realized by HyperLTL synthesis can, however, be much more sophisticated than the observational determinism needed for distributed synthesis. Consider, for example, the *dining cryptographers* problem [6]: three cryptographers $C_a,C_b,$ and $C_c$ sit at a table in a restaurant having dinner and either one of the cryptographers or, alternatively, the NSA must pay for their meal. Is there a protocol where each cryptographer can find out whether it was a cryptographer who paid or the NSA, but cannot find out which cryptographer paid the bill?

Synthesis from LTL formulas is known to be decidable in doubly exponential time [39]. The fact that the distributed synthesis problem is undecidable [40] immediately eliminates the hope for a similar general result for HyperLTL. However, since LTL is obviously a fragment of HyperLTL, this immediately leads to the question whether the synthesis problem is still decidable for fragments of HyperLTL that are close to LTL but go beyond LTL: when exactly does the synthesis problem become undecidable? From a more practical point of view, the interesting question is whether semi-algorithms for distributed synthesis [16, 28], which have been successful in constructing distributed systems from LTL specifications despite the undecidability of the general problem, can be extended to HyperLTL?

In this paper, we answer the first question by studying the $\exists ^*$, $\exists ^*\forall ^1$, and the ${ linear }\;\forall ^*$ fragment. We show that the synthesis problem for all three fragments is decidable, and the problem becomes undecidable as soon as we go beyond these fragments. In particular, the synthesis problem for the full $\forall ^*$ fragment, which includes observational determinism, is undecidable.

We answer the second question by studying the *bounded* version of the synthesis problem for the $\forall ^*$ fragment. In order to detect realizability, we ask whether, for a universal HyperLTL formula $\varphi$ and a given bound *n* on the number of states, there exists a representation of the strategy tree as a finite-state machine with no more than *n* states that satisfies $\varphi$. To detect unrealizability, we check whether there exists a counterexample to realizability of bounded size. We show that both checks can be effectively reduced to SMT solving.

### Related work

$\text {HyperLTL}$ [8] is a successor of the temporal logic $\text {SecLTL}$ [14] used to characterize temporal information flow. The model-checking [8, 25, 26], satisfiability [18, 19, 21], monitoring problem [1–4, 22–24, 33, 34], and the first-order extension [31] of $\text {HyperLTL}$ have been studied before. In [11], it has been shown that existential quantification in a $\text {HyperLTL}$ formula can be reduced to strategic choice. An extensive study of the hierarchy of hyperlogics beyond $\text {HyperLTL}$ has been initiated in [10].

We base our algorithms on well-known synthesis algorithms such as bounded synthesis [28] that itself is an instance of Safraless synthesis [36] for $\omega$-regular languages. A further technique that we adapt for hyperproperties is the bounded unrealizability method [29, 30].

Hyperproperties [9] can be seen as a unifying framework for many different properties of interest in multiple distinct areas of research. Information-flow properties in security and privacy research are hyperproperties [8]. $\text {HyperLTL}$ subsumes logics that reason over knowledge [8]. Information flow in distributed systems is another example of hyperproperties, and the $\text {HyperLTL}$ realizability problem subsumes both the distributed synthesis problem [27, 40] as well as synthesis of fault-tolerant systems [30]. In circuit verification, the semantic independence of circuit output signals on a certain set of inputs, enabling a range of potential optimizations, is a hyperproperty.

This article is an extended version of [20], including previously omitted proofs. Additionally, we show that HyperLTL realizability extends many previous extensions to LTL realizability, including realizability under incomplete information [35], distributed synthesis [27, 40], and fault-tolerant synthesis [30].

### Structure of this article

We introduce HyperLTL and necessary preliminaries in Sect. 2. In Sect. 3 we define the realizability problem for HyperLTL and demonstrate the expressiveness compared to classical extensions of LTL realizability. In the following section, we investigate the decidability for the realizability problem, where we characterize fragments based on the quantifier prefix. In Sects. 5 and 6 we give algorithms for the bounded realizability and unrealizability problem of universal HyperLTL, i.e., we bound the size of the system and environment, respectively, in order to derive a semi-decision procedure. We report on experimental evaluation of our prototype synthesis tool on a variety of benchmarks, involving distributed architectures, fault-tolerance, and secrecy properties.

## Preliminaries

### HyperLTL

$\text {HyperLTL}$ [8] is a temporal logic for specifying hyperproperties. It extends $\text {LTL}$ by quantification over trace variables $\pi$ and a method to link atomic propositions to specific traces. The set of trace variables is ${\mathcal {V}}$. Formulas in $\text {HyperLTL}$ are given by the grammarwhere $a \in \text {AP}$ and $\pi \in {\mathcal {V}}$. The alphabet of a $\text {HyperLTL}$ formula is $2^\text {AP}$. We allow the standard boolean connectives $\wedge$, $\rightarrow$, $\leftrightarrow$ as well as the derived $\text {LTL}$ operators release , eventually , globally , and weak until .

The semantics is given by the satisfaction relation $\vDash _T$ over a set of traces $T \subseteq (2^\text {AP})^\omega$. We define an assignment $\varPi : {\mathcal {V}}\rightarrow (2^\text {AP})^\omega$ that maps trace variables to traces. $\varPi [i,\infty ]$ is the trace assignment that for every $\pi$ maps $\varPi$ to the trace $\varPi (\pi )[i,\infty ]$, i.e., it removes the first *i* items from the traces.We write $T \vDash \varphi$ for $\{\} \vDash _T \varphi$ where $\{\}$ denotes the empty assignment. Two $\text {HyperLTL}$ formulas $\varphi$ and $\psi$ are equivalent, written $\varphi \equiv \psi$ if they have the same models. A $\text {HyperLTL}$ formula $\varphi$ is denoted satisfiable if there is a set of traces *T* which satisfies $\varphi$, i.e., $T \vDash \varphi$. The satisfiability problem is undecidable for general $\text {HyperLTL}$ formulas but becomes decidable if we renounce $\forall ^*\exists ^*$ formulas which alternate the quantifier from $\forall$ to $\exists$ [18]. For an $\text {LTL}$ formula $\varphi$, we denote by $\varphi [\pi ]$ the quantifier-free $\text {HyperLTL}$ formula where every proposition *a* is replaced by $a_\pi$.

*(In)dependence* is a hyperproperty that we will use multiple times in this article, thus, we define the following syntactic sugar. Given two disjoint subsets of atomic propositions $C \subseteq \text {AP}$ and $A \subseteq \text {AP}$, we define independence as the following $\text {HyperLTL}$ formula1which requires that the valuations of propositions *C* on traces $\pi$ and $\pi '$ have to be equal until and including the point in time where there is a difference in the valuation of some proposition in *A*. Prefacing universal quantification, that is, the formula $\forall \pi \forall \pi ' \mathpunct {.}D^{\pi ,\pi '}_{A \mapsto C}$ guarantees that every proposition $c \in C$ solely depends on propositions in *A*.

### Strategies

A *strategy*$$f :(2^{I})^* \rightarrow 2^{O}$$ maps sequences of input valuations $$2^{I}$$ to an output valuation $$2^{O}$$. The behavior of a strategy $$f:(2^{I})^* \rightarrow 2^{O}$$ is characterized by an infinite tree, called *computation tree*, that branches by the valuations of *I* and whose nodes $$w \in (2^{I})^*$$ are labeled with the strategic choice *f*(*w*). For an infinite word $$w = w_0 w_1 w_2 \cdots \in (2^{I})^\omega $$, the corresponding *trace* is defined as $$(f(\epsilon ) \cup w_0)(f(w_0) \cup w_1)(f(w_0 w_1) \cup w_2)\cdots \in (2^{I \cup O})^\omega $$. We lift the set containment operator $$\in $$ to the containment of a trace $$w = w_0 w_1 w_2 \cdots \in (2^{I \cup O})^\omega $$ in a strategy tree induced by $$f :(2^{I})^* \rightarrow 2^{O}$$, i.e., $$w \in f$$ if, and only if, $$f(\epsilon ) = w_0 \cap O$$ and $$f((w_0 \cap I) \cdots (w_i \cap I)) = w_{i+1} \cap O$$ for all $$i \ge 0$$. The set of traces produced by *f*, written $$ traces (f)$$, is $$\{w \mid w \in f\}$$. We define the satisfaction of a $$\text {HyperLTL}$$ formula $$\varphi $$ (over propositions $$I \cup O$$) on strategy *f*, written $$f \vDash \varphi $$, as $$ traces (f) \vDash \varphi $$. Thus, a strategy *f* is a model of $$\varphi $$ if the set of traces of *f* is a model of $$\varphi $$.

## HyperLTL synthesis

In this section, we introduce the realizability problem for $\text {HyperLTL}$ and compare its expressiveness to various previous extensions of the $\text {LTL}$ realizability problem.

### Definition 1

($\text {HyperLTL}$*Realizability*) A $\text {HyperLTL}$ formula $\varphi$ over atomic propositions $\text {AP}= I \mathbin {{{\dot{\cup }}}}O$ is realizable if there is a strategy $f :(2^{I})^* \rightarrow 2^{O}$ that satisfies $\varphi$.

The fragment of $\text {HyperLTL}$ with only a single, universal quantifier $\forall \pi \mathpunct {.}\varphi$ is equivalent to the $\text {LTL}$ realizability problem of $\varphi$. With two universal quantifiers, one can express relations between traces in the execution tree, thus, one can express the $\text {LTL}$ realizability problem with restricted information flow like incomplete information [35], distributed synthesis [27, 40], and fault-tolerant synthesis [13, 30].

### Incomplete information

The realizability problem with incomplete information [35] is a tuple ${\langle \varphi , I, O, H \rangle }$, where $\varphi$ is an $\text {LTL}$ formula, *I* is a set of input propositions, *O* is a set of output propositions, and $H \subseteq I$ is a set of hidden inputs not observable by the system. Thus, a realizing strategy $f :(2^{I {\setminus } H})^* \rightarrow 2^{O}$ has a computation tree that only branches by $I {\setminus } H$. In order to evaluate $\varphi$, which may include propositions *H*, the computation tree is *widened* [35] by *H*. In $\text {HyperLTL}$, we can verify that a strategy $f' :(2^{I})^* \rightarrow 2^{O}$ has the same output-behavior as a *H*-widened strategy *f* by checking $f' \vDash \forall \pi \forall \pi ' \mathpunct {.}D^{\pi ,\pi '}_{I {\setminus } H \mapsto O}$.

#### Theorem 1

The $\text {HyperLTL}$ realizability problem subsumes the $\text {LTL}$ realizability with incomplete information problem.

#### Proof

Given ${\langle \varphi , I, O, H \rangle }$, the following $\text {HyperLTL}$ formula over inputs *I* and outputs *O* is equirealizable.

$$\begin{aligned} \forall \pi \forall \pi ' \mathpunct {.}\varphi [\pi ] \wedge D^{\pi ,\pi '}_{I {\setminus } H \mapsto O} \end{aligned}$$

$$\square $$

### Distributed synthesis

The distributed synthesis problem was introduced by Pnueli and Rosner [40] and introduces the concept of *architectures* as a constraint on the information flow. An architecture is a set of processes *P*, with distinct environment process ${p_\textit{env}}\in P$, such that the processes produce outputs synchronously, but each process bases its decision only on the history of valuation of inputs that it observes.

Formally, a distributed architecture ${\mathcal {A}}$ is a tuple ${\langle P, p_{env}, {\mathcal {I}}, {\mathcal {O}} \rangle }$ where *P* is a finite set of processes with distinguished environment process $p_\textit{env} \in P$. The functions ${\mathcal {I}} :P \rightarrow 2^{\text {AP}}$ and ${\mathcal {O}} :P \rightarrow 2^{\text {AP}}$ define the inputs and outputs of processes. While processes may share the same inputs (in case of broadcasting), the outputs of processes must be pairwise disjoint, i.e., for all $p \ne p' \in P$ it holds that ${\mathcal {O}}(p) \cap {\mathcal {O}}(p') = \emptyset$. W.l.o.g. we assume that ${\mathcal {I}}({p_\textit{env}}) = \emptyset$. We denote by $P^{-}= P {\setminus } \{{p_\textit{env}}\}$ the set of processes excluding the environment process.

**Fig. 1:**
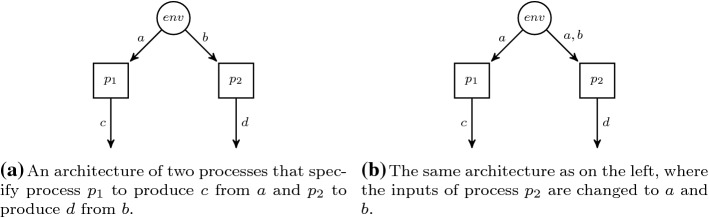
Distributed architectures

The distributed realizability problem for architectures without *information forks* [27] is decidable. Intuitively, an information fork is a situation where two distinct processes $p, p' \in P$ receive environment inputs *I* and $I'$ (may be transitive through other processes) such that both observe inputs that the other process does not observe, i.e., there exist $i \in I$ and $i' \in I'$ such that $i \notin I'$ and $i' \notin I$. We depict two example architectures in Fig. 1. The architecture in Fig. 1a contains an information fork while the architecture in Fig. 1b does not. Furthermore, the processes in Fig. 1b can be ordered linearly according to the subset relation on the inputs.

#### Theorem 2

The $\text {HyperLTL}$ realizability problem subsumes the distributed $\text {LTL}$ realizability problem.

#### Proof

Given a distributed realizability problem ${\langle \varphi ,{\mathcal {A}} \rangle }$, the following $\text {HyperLTL}$ formula over inputs ${\mathcal {O}}({p_\textit{env}})$ for $P^{-}$ and outputs $\bigcup _{p \in P^{-}} {\mathcal {O}}(p)$ is equirealizable:

$$\begin{aligned} \forall \pi \forall \pi ' \mathpunct {.}\varphi [\pi ] \wedge \bigwedge _{p \in P^{-}} D^{\pi ,\pi '}_{{\mathcal {I}}(p) \mapsto {\mathcal {O}}(p)} \end{aligned}$$

$$\square $$

### Asynchronous distributed synthesis

The asynchronous system model [42] is a generalization of the synchronous model discussed previously. In this model, we have a global scheduler, controlled by the environment, that decides when and which processes are scheduled. The resulting distributed realizability problem is already undecidable for $\text {LTL}$ specifications and systems with more than one process [42].

#### Theorem 3

The $\text {HyperLTL}$ realizability problem subsumes the asynchronous distributed $\text {LTL}$ realizability problem.

#### Proof

Let $${\mathcal {A}}= {\langle P, p_{env}, {\mathcal {I}}, {\mathcal {O}} \rangle }$$ be a distributed architecture. To model scheduling, we introduce an additional set $$ Sched = \{ sched _p \mid p \in P^{-}\}$$ of atomic propositions. The valuation of $$ sched _p$$ indicates whether system process *p* is currently scheduled or not. A process $$p \in P^{-}$$ may observe whether it is scheduled or not, that is, it may depend on $$ sched _p$$. The environment can decide at every step which processes to schedule. When a process is not scheduled, its output behavior does not change [28]. As the scheduling is controlled by the environment, we assume that every process is infinitely often scheduled, as otherwise, the environment wins by simply not scheduling any process.

Given an asynchronous distributed realizability problem ${\langle \varphi ,{\mathcal {A}} \rangle }$, the following $\text {HyperLTL}$ formula over inputs ${\mathcal {O}}({p_\textit{env}}) \cup Sched$ and outputs $\bigcup _{p \in P^{-}} {\mathcal {O}}_p$ is equirealizable:$\square$

### Symmetric synthesis

A special case of distributed synthesis is symmetric synthesis [15], which, additionally to distributivity, requires that all system processes act exactly the same if they are given the same inputs. Formally, symmetric synthesis requires a symmetric architecture ${\langle P, p_{env}, {\mathcal {I}}, {\mathcal {O}} \rangle }$ where for each process $p \in P^{-}$, ${|{\mathcal {I}}(p)|} = n$ and ${|{\mathcal {O}}(p)|} = m$ for some $n,m \in {\mathbb {N}}$. We assume an implicit ordering of inputs and output per process and use the notation ${\mathcal {I}}(p)_j$ and ${\mathcal {O}}(p)_j$ to access the *j*-th input and output of process $p \in P^{-}$, respectively. Then, we can express the symmetry constraint as an $\text {LTL}$ formula 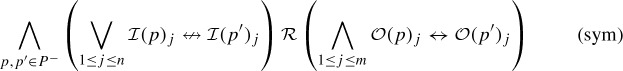


#### Theorem 4

The $\text {HyperLTL}$ realizability problem subsumes the symmetric (distributed) $\text {LTL}$ realizability problem.

#### Proof

Given a symmetric realizability problem over architecture ${\mathcal {A}}$ and specifications $\varphi _1,\dots , \varphi _k$ for the *k* processes the following $\text {HyperLTL}$ formula over inputs ${\mathcal {O}}({p_\textit{env}})$ for $P^{-}$ and outputs $\bigcup _{p \in P^{-}} {\mathcal {O}}(p)$ is equirealizable:

$$\begin{aligned} \forall \pi \forall \pi ' \mathpunct {.}\bigwedge _{1 \le i \le k} \varphi _i[\pi ] \wedge \bigwedge _{p \in P^{-}} D^{\pi ,\pi '}_{{\mathcal {I}}(p) \mapsto {\mathcal {O}}(p)} \wedge ~sym[\pi ] \end{aligned}$$

$$\square $$

### Fault-tolerant synthesis

We consider another extension to the distributed synthesis problem where we incorporate the possibility that communication *between* processes may be subject to faults, such as Byzantine faults [29, 30]. In the distributed synthesis formulation above, communication from some process *p* to $p'$ was encoded as an atomic proposition *a* such that $a \in {\mathcal {O}}(p)$ and $a \in {\mathcal {I}}(p')$. In the fault-tolerance encoding, we split this connection into a sending part $a_s \in {\mathcal {O}}(p)$ and a receiving part $a_r \in {\mathcal {I}}(p')$ where $a_r \in {\mathcal {O}}({p_\textit{env}})$ is a proposition controlled by the environment. To relate $a_s$ and $a_r$, we add the assumption  to the $\text {LTL}$ specification. This encoding uses more atomic propositions and additional $\text {LTL}$ constraints but is otherwise equivalent to the one presented before.

This increased flexibility, that is, being able to specify communication using temporal logic, allows us to express unreliable communication. For example, using the assumption  specifies a delay of one time step on the receiver,  specifies a stuck-at-one fault, and $\top$ specifies a Byzantine fault where the environment takes over the communication. This alone is not enough though: if a process gets such a specification it knows which receiving propositions present actual values and which one is subject to a fault. Thus, the processes are challenged in *multiple architectures*, where each architecture may have a different set of communication faults as well as specifications: depending on the type of failure, the overall system may only be expected to satisfy a weaker property then the original, non-faulty one.

Formally, the fault-tolerant realizability problem is a tuple ${\langle {\mathcal {A}}, \varphi _1, \dots , \varphi _n \rangle }$, where ${\mathcal {A}}$ is a distributed architecture with the property that every process receives only environment inputs, i.e., ${\mathcal {I}}(p) \subseteq {\mathcal {O}}({p_\textit{env}})$ for all $p \in P^{-}$, and $\varphi _1, \dots , \varphi _n$ are $\text {LTL}$ formulas. For Byzantine fault-tolerance,  where $R_i \subseteq O \times I$ are the non-faulty communication of architecture *i* and $\psi _i$ is the $\text {LTL}$ specification that should be ensured.

**Fig. 2:**
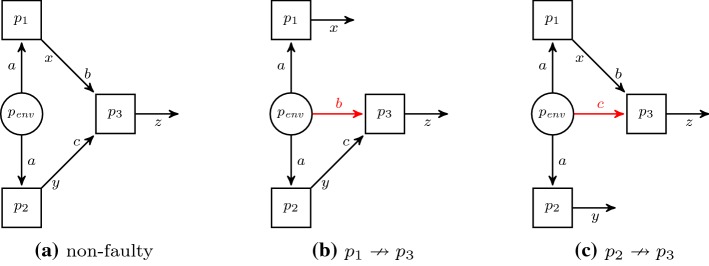
Visual interpretation of a fault-tolerance specification: on the left is the original (non-faulty) architecture where the communication between $p_1 \rightarrow p_3$ and $p_2 \rightarrow p_3$ is intact. The two architectures on the right represent the case where either $p_1 \nrightarrow p_3$ or $p_2 \nrightarrow p_3$. In this case, the receiving propositions *b* and *c*, respectively, are controlled by the environment. In fault-tolerant synthesis, we search for strategies for processes $p_1$, $p_2$, and $p_3$ such that the specification is satisfied in all architectures

As an example, consider the architecture

2 $$\begin{aligned} \langle \{{p_\textit{env}}, p_1, p_2, p_3\}, {p_\textit{env}}, \{p_1 \mapsto \{a\}, p_2 \mapsto \{a\}, p_3 \mapsto \{b,c\}\},\nonumber \\ \{{p_\textit{env}}\mapsto \{a,b,c\}, p_1 \mapsto \{x\}, p_2 \mapsto \{y\}, p_3 \mapsto \{z\}\} \rangle \end{aligned}$$

with specifications , , and . This example specification asserts that $\psi$ holds in all three architectures depicted in Fig. 2, i.e., if either $p_1 \xrightarrow {x} p_3$ or $p_2 \xrightarrow {y} p_3$ fails, but not both of them. Hence, process $p_3$ cannot know whether the information given via propositions *b* or *c* is correct.

#### Theorem 5

The $\text {HyperLTL}$ realizability problem subsumes the fault-tolerant $\text {LTL}$ realizability problem.

#### Proof

Given ${\langle {\mathcal {A}}, \varphi _1, \dots , \varphi _n \rangle }$, the following $\text {HyperLTL}$ formula over inputs ${\mathcal {O}}({p_\textit{env}})$ and outputs $\bigcup _{p \in P^{-}} {\mathcal {O}}_p$ is equirealizable:

$$\begin{aligned} \forall \pi \forall \pi ' \mathpunct {.}\bigwedge _{1 \le i \le n} \varphi _i[\pi ] \wedge \bigwedge _{p \in P^{-}} D^{\pi ,\pi '}_{{\mathcal {I}}(p) \mapsto {\mathcal {O}}(p)} \end{aligned}$$

$$\square $$

## Deciding $\text {HyperLTL}$ synthesis

In this section, we identify fragments of $\text {HyperLTL}$ for which the realizability problem is decidable. Our findings are summarized in Table 1.

**Table 1:** Summary of decidability results

$\exists ^*$	$\forall ^*$	${ linear }\;\forall ^*$	$\exists ^* \forall ^1$	$\exists ^* \forall ^{>1}$	$\forall ^* \exists ^*$
$\textsc {PSpace}$-complete	Undecidable	Non-elem.	$\textsc {3ExpTime}$	Undecidable	Undecidable

We base our investigation on the structure of the quantifier prefix of the HyperLTL formulas. We call a HyperLTL formula $\varphi$ (quantifier) *alternation-free* if the quantifier prefix consists solely of either universal or existential quantifiers. We denote the corresponding fragments as the (universal) $\forall ^*$ and the (existential) $\exists ^*$ fragment, respectively. A HyperLTL formula is in the $\exists ^* \forall ^*$ fragment, if it starts with arbitrarily many existential quantifiers, followed by arbitrarily many universal quantifiers. Analogously for the $\forall ^*\exists ^*$ fragment. For a given natural number *n*, we refer to a bounded number of quantifiers with $\forall ^n$, respectively $\exists ^n$. The $\forall ^1$ realizability problem is equivalent to the $\text {LTL}$ realizability problem.

### $\exists ^*$ Fragment

We show that the realizability problem for existential $\text {HyperLTL}$ is $\textsc {PSpace}$-complete. We reduce the realizability problem to the satisfiability problem for bounded one-alternating $\exists ^*\forall ^2 \text {HyperLTL}$ [18], i.e., finding a trace set *T* such that $T \vDash \varphi$.

#### Lemma 1

An existential $\text {HyperLTL}$ formula $\varphi = \exists \pi _1 \cdots \exists \pi _n \mathpunct {.}\psi$ is realizable if, and only if, $\varphi _{ sat } :=\exists \pi _1 \cdots \exists \pi _n \mathpunct {.}\forall \pi \forall \pi ' \mathpunct {.}\psi \wedge D^{\pi ,\pi '}_{I \mapsto O}$ is satisfiable.

#### Proof

Assume $f :(2^{I})^* \rightarrow 2^{O}$ realizes $\varphi$, that is $f \vDash \varphi$. Let $T = traces (f)$ be the set of traces generated by *f*. It holds that $T \vDash \varphi$ and $T \vDash \forall \pi ,\pi ' \mathpunct {.}D^{\pi ,\pi '}_{I \mapsto O}$. Therefore, *T* witnesses the satisfiability of $\varphi _{ sat }$. For the reverse direction, assume that $\varphi _{ sat }$ is satisfiable. Let *S* be a set of traces that satisfies $\varphi _{ sat }$. We construct a strategy $f :(2^{I})^* \rightarrow 2^{O}$ as

$$\begin{aligned} f(\sigma ) = {\left\{ \begin{array}{ll} w_{|\sigma |} \cap O &{}\quad \text {if } \sigma \text { is a prefix of some } w|_I \text { with } w \in S\text {, and} \\ \emptyset &{}\quad \text {otherwise}. \end{array}\right. } \end{aligned}$$

where $w|_I$ denotes the trace restricted to *I*, meaning that $w_i \cap I$ for all $i \ge 0$. Note that if there are multiple candidates $w \in S$, then $w_{|\sigma |} \cap O$ is equal across all of them due to the required determinism $\forall \pi \forall \pi ' \mathpunct {.}D^{\pi ,\pi '}_{I \mapsto O}$. By construction, all traces in *S* are contained in *f*, and together with $S \vDash \varphi$, it holds that $f \vDash \varphi$ as the sets of sets of traces satisfying the existential formula $\varphi$ are upward closed. $\square$

#### Theorem 6

Realizability of existential $\text {HyperLTL}$ specifications is decidable.

#### Proof

The formula $\varphi _{ sat }$ from Lemma 1 is in the $\exists ^*\forall ^2$ fragment, for which satisfiability is decidable [18]. $\square$

#### Corollary 1

Realizability of existential $\text {HyperLTL}$ specifications is $\textsc {PSpace}$-complete.

#### Proof

Given an existential $\text {HyperLTL}$ formula, we gave a linear reduction to the satisfiability of the $\exists ^*\forall ^2$ fragment in Lemma 1. The satisfiability problem for a bounded number of universal quantifiers is in $\textsc {PSpace}$ [18]. Hardness follows from $\text {LTL}$ satisfiability [43], which is equivalent to the $\exists ^1$ fragment. $\square$

### $\forall ^*$ Fragment

In the following, we will use the *distributed synthesis* problem defined before, i.e., the problem whether there is an implementation of processes in a distributed architecture (cf. Fig. 1) that satisfies an $\text {LTL}$ formula.

#### Theorem 7

The synthesis problem for universal $\text {HyperLTL}$ becomes undecidable as soon as we have more than one universal quantifier.

#### Proof

In the $\forall ^*$ fragment (and thus in the $\exists ^*\forall ^*$ fragment), we can encode a distributed architecture [40], for which $\text {LTL}$ synthesis is undecidable. In particular, we can encode the architecture shown in Fig. 1a. This architecture basically specifies *c* to depend only on *a* and analogously *d* on *b*. That can be encoded by $D^{\pi ,\pi '}_{\{a\} \mapsto \{c\}}$ and $D^{\pi ,\pi '}_{\{b\} \mapsto \{d\}}$. The $\text {LTL}$ synthesis problem for this architecture is undecidable [40], i.e., given an $\text {LTL}$ formula over $I=\{a,b\}$ and $O=\{c,d\}$, we cannot automatically construct processes $p_1$ and $p_2$ that realize the formula. $\square$

### Linear $\forall ^*$ fragment

As we have already seen in Theorem 2, we can represent distributed architectures in HyperLTL by using the dependency formulas $D^{\pi ,\pi '}_{I \mapsto O}$. In this section, we do the reverse direction. Given a HyperLTL formula $\varphi$, we try to find an equivalent formula which has the distributed form $\forall \pi \mathpunct {.}\forall \pi '\mathpunct {.}\varphi ' \wedge (\textit{dep})$ where $\varphi '$ only uses the path variable $\pi$ and where $(\textit{dep})$ is a conjunction of dependency formulas. These formulas can then be translated to distributed architectures on which we can use the already existent techniques to deal with the synthesis problem. In particular, we can check whether the resulting distributed synthesis problem has an information fork. If this is not the case, the problem is decidable [27].

For finding the distributed form, we need to find the “LTL-part” $\varphi '$ of the HyperLTL property $\varphi$. We call this *collapsing*$\varphi$ to $\varphi '$. This transformation collapses the universal quantifier into a single one and renames the path variables accordingly. For example,  is transformed into an equivalent $\forall ^1$ formula . However, this transformation does not always produce equivalent formulas as  is not equivalent to its collapsed form . Let $\varphi$ be $\forall \pi _1 \cdots \forall \pi _n \mathpunct {.}\psi$. We define the collapsed formula of $\varphi$ as $collapse (\varphi ) :=\forall \pi \mathpunct {.}\psi [\pi _1 \mapsto \pi ][\pi _2 \mapsto \pi ]\dots [\pi _n \mapsto \pi ]$ where $\psi [\pi _i \mapsto \pi ]$ replaces all occurrences of $\pi _i$ in $\psi$ with $\pi$. Although the collapsed term is not always equivalent to the original formula, we can use it as an indicator whether it is possible at all to express a universal formula with only one quantifier as stated in the following lemma.

#### Lemma 2

Either $\varphi \equiv collapse (\varphi )$ or $\varphi$ has no equivalent $\forall ^1$ formula.

#### Proof

Suppose there is some $\psi \in \forall ^1$ with $\psi \equiv \varphi$. We show that $\psi \equiv collapse (\varphi )$. Let *T* be an arbitrary set of traces. Let ${\mathcal {T}} = \{\{w\} \mid w \in T \}$. Because $\psi \in \forall ^1$, $T \vDash \psi$ is equivalent to $\forall T' \in {\mathcal {T}} \mathpunct {.}T' \vDash \psi$, which is by assumption equivalent to $\forall T' \in {\mathcal {T}} \mathpunct {.}T' \vDash \varphi$. Now, $\varphi$ operates on singleton trace sets only. This means that all quantified paths have to be the same, which yields that we can use the same path variable for all of them. So $\forall T' \in {\mathcal {T}} \mathpunct {.}T' \vDash \varphi \leftrightarrow T' \vDash collapse (\varphi )$ that is again equivalent to $T \vDash collapse (\varphi )$. Because $\psi \equiv collapse (\varphi )$ and $\psi \equiv \varphi$ it holds that $\varphi \equiv collapse (\varphi )$. $\square$

Now that we have the $\varphi '$-part of the distributive form, we need to find the variable dependencies. More precisely, given a formula $\varphi$, we seek for variable dependencies of the form $D^{\pi ,\pi '}_{J \mapsto \{o\}}$ with $J\subseteq I$ for every output $o \in O$. These $J's$ can be brute-forced. For doing so, we just check for each case the equivalence between $\varphi$ and $\forall \pi \mathpunct {.}\forall \pi '\mathpunct {.} collapse (\varphi )\wedge (\textit{dep})$ where (*dep*) is the conjunction of all $D^{\pi ,\pi '}_{J \mapsto \{o\}}$. If this is the case and furthermore, the $D^{\pi ,\pi '}_{J_i \mapsto \{o_i\}}$ constraints can be ordered such that $J_i\subseteq J_{i+1}$ for all *i*, we have a linear architecture. Linear architectures are architectures without information fork. Thus, they are decidable. We define the linear fragment to encompass exactly these linear architectures. All in all, there are three steps to check whether $\varphi$ is in the linear fragment:

1. First, we have to add input-determinism to the formula $\varphi _{ det } :=\forall \pi \mathpunct {.}\forall \pi '\mathpunct {.}\varphi \wedge D^{\pi ,\pi '}_{I \mapsto O}$. This preserves realizability as strategies are input-deterministic.
2. Find for each output variable $o_i \in O$ possible sets of variables $J_i$ on which the $o_i$ depend, such that $J_i\subseteq J_{i+1}$. To check whether the choice of *J*’s is correct, we test if $\forall \pi \mathpunct {.}\forall \pi '\mathpunct {.} collapse (\varphi ) \wedge \bigwedge _{o_i \in O} D^{\pi ,\pi '}_{J_i \mapsto \{o_i\}}$ is equivalent to $\varphi _{ det }$. This equivalence check is decidable as both formulas are in the universal fragment [18].
3. Finally, we construct the corresponding distributed realizability problem with linear architecture. We define the distributed architecture ${\mathcal {A}}= {\langle P, p_{env},{\mathcal {I}},{\mathcal {O}} \rangle }$ with $P = \{p_i \mid o_i \in O\} \cup \{{p_\textit{env}}\}$, ${\mathcal {I}}(p_i) = J_i$, ${\mathcal {O}}(p_i) = \{o_i\}$, and ${\mathcal {O}}({p_\textit{env}}) = I$. The $\text {LTL}$ specification for the distributed synthesis problem is $collapse (\varphi )$

#### Definition 2

*(linear fragment of*$\forall ^*$*)* In the context of a synthesis problem with inputs *I* and outputs *O*, a formula $\varphi$ is in the linear fragment of $\forall ^*$ iff for all $o_i \in O$ there is a $J_i \subseteq I$ such that $\forall \pi \mathpunct {.}\forall \pi '\mathpunct {.}\varphi \wedge D^{\pi ,\pi '}_{I \mapsto O} \equiv \forall \pi \mathpunct {.}\forall \pi '\mathpunct {.} collapse (\varphi ) \wedge \bigwedge _{o_i \in O} D^{\pi ,\pi '}_{J_i \mapsto \{o_i\}}$ and $J_i \subseteq J_{i+1}$ for all *i*.

Note that each $\forall ^1$ formula $\varphi$ (or $\varphi$ is collapsible to a $\forall ^1$ formula) is in the linear fragment because we can set all $J_i=I$ and additionally $collapse (\varphi )=\varphi$ holds.

An example of a formula in the linear fragment of $\forall ^*$ is  with $I=\{a,b\}$ and $O=\{c,d,e\}$. The corresponding formula asserting input-deterministism is $\forall \pi \mathpunct {.}\forall \pi '\mathpunct {.}\varphi _{det}=\varphi \wedge D^{\pi ,\pi '}_{I \mapsto O}$. One possible choice of *J*’s is $\{a,b\}$ for *c*, $\{a\}$ for *d* and $\{a,b\}$ for *e*. Note, that one can use either $\{a,b\}$ or $\{a\}$ for *c* as $\forall \pi \mathpunct {.}\forall \pi '\mathpunct {.}D^{\pi ,\pi '}_{\{a\} \mapsto \{d\}} \wedge (c_\pi \leftrightarrow d_\pi )$ implies $\forall \pi \mathpunct {.}\forall \pi '\mathpunct {.}D^{\pi ,\pi '}_{\{a\} \mapsto \{c\}}$. However, the apparent alternative $\{b\}$ for *e* would yield an undecidable architecture with information fork. It holds that $\varphi _{det}$ and $\forall \pi \mathpunct {.}\forall \pi '\mathpunct {.} collapse (\varphi ) \wedge D^{\pi ,\pi '}_{\{a,b\} \mapsto \{c\}} \wedge D^{\pi ,\pi '}_{\{a\} \mapsto \{d\}} \wedge D^{\pi ,\pi '}_{\{a,b\} \mapsto \{e\}}$ are equivalent and, thus, that $\varphi$ is in the linear fragment.

#### Theorem 8

The realizability of the linear fragment of $\text {HyperLTL}$ is decidable.

#### Proof

It holds that $\forall \pi \mathpunct {.}\forall \pi '\mathpunct {.}\varphi \wedge D^{\pi ,\pi '}_{I \mapsto O} \equiv \forall \pi \mathpunct {.}\forall \pi '\mathpunct {.} collapse (\varphi ) \wedge \bigwedge _{o_i \in O} D^{\pi ,\pi '}_{J_i \mapsto \{o_i\}}$ for some $J_i$’s. The $\text {LTL}$ distributed realizability problem for $collapse (\varphi )$ in the constructed architecture *A* is equivalent to the $\text {HyperLTL}$ realizability of $\varphi$ as the architecture ${\mathcal {A}}$ represents exactly the input-determinism represented by formula $\bigwedge _{o_i \in O} D^{\pi ,\pi '}_{J_i \mapsto \{o_i\}}$. The architecture is linear and, thus, the realizability problem is decidable. $\square$

#### Corollary 2

The realizability problem of the linear fragment of $\text {HyperLTL}$ can be checked in non-elementary time.

The reason for this is that solving the distributed synthesis problem takes non-elementary time in the amount of variables. Surprisingly, this runtime is not (more than linearly) dependent on the amount of quantifiers as they are directly reduced to two.

### $\exists ^* \forall ^1$ Fragment

In this fragment, we consider arbitrary many existential trace quantifiers followed by a single universal trace quantifier. This fragment turns out to be still decidable. We solve the realizability problem for this fragment by reducing it to a decidable fragment of the distributed realizability problem for $\text {LTL}$.

#### Theorem 9

Realizability of $\exists ^* \forall ^1 \text {HyperLTL}$ specifications is decidable.

#### Proof

**Fig. 3:**
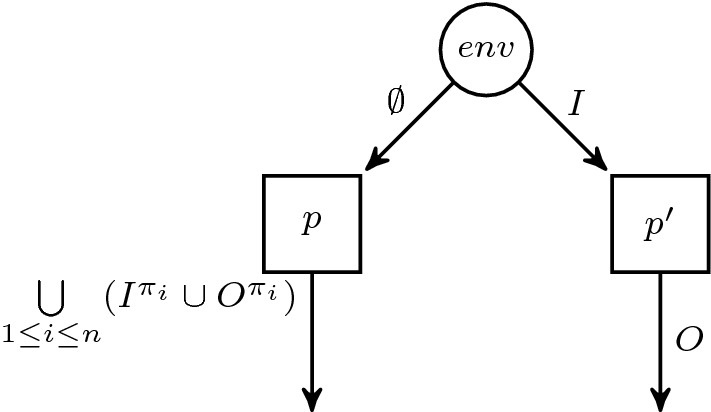
Visualization of the architecture used in the $\exists ^*\forall ^1$ reduction in the proof of Theorem 9

Let $\varphi$ be $\exists \pi _1 \cdots \exists \pi _n \mathpunct {.}\forall \pi ' \mathpunct {.}\psi$. We reduce the realizability problem of $\varphi$ to the distributed realizability problem for $\text {LTL}$. Intuitively, we use a two-process distributed architecture where the first process *p* is supposed to produce the traces for the leading existential quantification and the second process $p'$ represents the realizing strategy. The architecture is depicted in Fig. 3.

For every existential trace quantifier $\pi$, we introduce a copy of every atomic proposition for the distributed realizability problem, written $a^{\pi }$ for $a \in \text {AP}$. We use the same notation for sets of atomic propositions, e.g., $I^{\pi } = \{i^\pi \mid i \in I\}$. Process *p* has no inputs, thus, produces only a single trace, and it controls the outputs $\bigcup \limits _{1 \le i \le n} (I^{\pi _i} \cup O^{\pi _i})$. Using an appropriate valuation of its outputs, process *p* selects the paths in the strategy tree corresponding to the existential trace quantifiers $\exists \pi _1 \cdots \exists \pi _n$. Thus, those output propositions of process *p* have to encode an actual path in the strategy tree produced by $p'$. To ensure this, we add the $\text {LTL}$ constraint  that asserts that if the inputs correspond to some path in the strategy tree, the outputs on those paths have to be the same. The resulting architecture ${\mathcal {A}}_\varphi$ is

$$\begin{aligned} \langle&\{{p_\textit{env}},p,p'\}, {p_\textit{env}}, \{ p \mapsto \emptyset , p' \mapsto I \}, \\&\{ {p_\textit{env}}\mapsto I, p \mapsto \bigcup _{1 \le i \le n} (I^{\pi _i} \cup O^{\pi _i}), p' \mapsto O\} \rangle . \end{aligned}$$

It is easy to verify that $${\mathcal {A}}_\varphi $$ does not contain an information fork, thus the realizability problem is decidable [27]. The $$\text {LTL}$$ specification $$\theta $$ is  where we replace every $$a_\pi $$ by $$a^\pi $$ for existential traces and $$a_{\pi '}$$ to *a* in $$\psi $$. The implementation of process $$p'$$ (if it exists) is a realizing strategy for the $$\text {HyperLTL}$$ formula (process *p* producing witnesses for the $$\exists $$ quantifiers): Assume that there are realizing strategies for $${\langle {\mathcal {A}}_\varphi ,\theta \rangle }$$, i.e., $$f_{p} :(2^{\emptyset })^* \rightarrow 2^{\bigcup _{1 \le i \le n} (I^{\pi _i} \cup O^{\pi _i})}$$ and $$f_{p'} :(2^{I})^* \rightarrow 2^{O}$$. $$f_{p'}$$ is a realizing strategy for $$\varphi $$ as well: By the HyperLTL semantics, we have to show that there is a trace assignment $$\varPi :{\mathcal {V}}_\exists \rightarrow traces (f_{p'})$$ such that for all $$t \in traces (f_{p'})$$ it holds that $$\varPi [\pi ' \rightarrow t] \vDash _{ traces (f_{p'})} \psi $$. We define $$\varPi $$ in the following. Note that $$ traces (f_p)$$ is a singleton set and let $$t_p \in \left( 2^{\bigcup _{1 \le i \le n} (I^{\pi _i} \cup O^{\pi _i})} \right) ^\omega $$ be the corresponding trace. For every $$\pi _i \in \{\pi _1,\ldots ,\pi _n\}$$, we define $$\varPi (\pi _i) = t_p|_{I^{\pi _i}}$$ where we replace $$a^{\pi _i}$$ by *a* for every $$a \in \text {AP}$$. This, together with $$\theta $$ shows that $$\psi $$ holds for every chosen path $$t \in traces (f_{p'})$$ for $$\pi '$$.

Conversely, a model for $\varphi$ can be used as an implementation of *p* and $p'$: Let $f :(2^{I})^* \rightarrow 2^{O}$ be a realizing strategy of $\varphi$. We use *f* as a strategy for $p'$. We construct the single trace produced by *p* using the existential trace assignment $\varPi :{\mathcal {V}}_\exists \rightarrow traces (f)$. Let $t_1,\ldots ,t_n \in traces (f)$ be the corresponding traces. We construct a single trace $t_p$ by replacing propositions $a \in \text {AP}$ by $a^{\pi _i}$ for every $t_i$ and the subsequent union of the resulting traces (which now have pairwise disjoint propositions). Due to the construction, $f_{p}$ satisfies  and thus, the distributed architecture satisfies $\theta$. Hence, the distributed synthesis problem ${\langle {\mathcal {A}}_\varphi ,\theta \rangle }$ has a solution if, and only if, $\varphi$ is realizable. $\square$

### $\forall ^* \exists ^*$ Fragment

The last fragment to consider are formulas in the $\forall ^*\exists ^*$ fragment. Whereas the $\exists ^*\forall ^1$ fragment remains decidable, the realizability problem of $\forall ^*\exists ^*$ turns out to be undecidable even when restricted to only one quantifier of both sorts ($\forall ^1\exists ^1$).

#### Theorem 10

Realizability of $\forall ^* \exists ^*$HyperLTL is undecidable.

#### Proof

We will prove this theorem by reducing Post’s Correspondence Problem (PCP) [41] to synthesizing an $\forall ^1\exists ^1$ formula. This proof follows the one from [18]. In PCP, we are given two lists $\alpha$ and $\beta$ of same length which consist of finite words from some alphabet $\varSigma$. For example, $\alpha$, with $\alpha _1 = a$, $\alpha _2 = ab$ and $\alpha _3 = bba$ and $\beta$, with $\beta _1 = baa$, $\beta _2 = aa$ and $\beta _3 = bb$. Here $\alpha _i$ denotes the *ith* element of the list, and $\alpha _{i,j}$ denotes the *jth* symbol of the *ith* element. In this example, $\alpha _{3,1}$ corresponds to *b*. PCP is the problem to find an index sequence $(i_k)_{1 \le k \le K}$ with $K \ge 1$ and $1 \le i_k \le n$ for all *k*, such that $\alpha _{i_1}\dots \alpha _{i_K}=\beta _{i_1}\dots \beta _{i_K}$. We denote the finite words of a PCP solution with $i_\alpha$ and $i_\beta$, respectively. It is a useful intuition to think of the PCP instance as a set of *n* domino stones. A possible solution for this PCP instance would be (3, 2, 3, 1), since this stone sequence produces the same word, i.e., $bbaabbbaa = i_\alpha = i_\beta$.

Let a PCP instance with $\varSigma = \{a_1,a_2,\ldots , a_n\}$ and two lists $\alpha$ and $\beta$ be given. We choose our set of atomic propositions as follows: ${ AP } := I\;{{\dot{\cup }}}\;O$ with $I := \{i\}$ and $O:= (\varSigma \cup \{{\dot{a}}_1, {\dot{a}}_2,\ldots ,{\dot{a}}_n\} \cup {\#})^2$, where we use the dot symbol to encode that a stone starts at this position of the trace. We write ${\tilde{a}}$ if we do not care if this symbol is an *a* or ${\dot{a}}$ and use $*$ as syntactic sugar for an arbitrary symbol of the alphabet.

We encode the PCP instance into a HyperLTL formula that is realizable if and only if the PCP instance has a solution as follows:

$$ \begin{aligned} \varphi _{ reduc } := \varphi _{ sol }(\pi ) \wedge \forall \pi \exists \pi '.\;\varphi _{ rel }(\pi ) \rightarrow&\varphi _{ is++ }(\pi ,\pi ')\\&{} \wedge \varphi _{ start }(\varphi _{ stone \& shift }(\pi ,\pi '),\pi ) \end{aligned}$$

. Here, we associate the left part of the pairs with the upper ($\alpha$) part of the domino stones and the right part with the lower ($\beta$) part of the domino stones. And only taking the left side of the pairs along the path $\pi$ we should get $i_\alpha$, respectively for $i_\beta$. Therefore, this formula essentially states that the path which has  encodes our *solution* and has $i_\alpha =i_\beta$. The $\#$ symbols at the end are placeholders for ensuring that the sequence has a finite size.
. This defines the set of *relevant* traces trough our strategy tree. They can be imagined being *parallel* to each other or thought of as the solution trace  but with some additional $\lnot i$ in front.
. This defines that a trace $\pi '$ is a successor of $\pi$. It essentially states that $\pi '$ has exactly one more $\lnot i$ at the beginning than $\pi$, i.e., it is the *next* parallel trace.
- Essentially, we now want $\pi '$ to be exactly $\pi$ but with the first stone removed and the rest shifted to the front. This can be best illustrated with the example from above. The full sequence at the trace  represents the solution with the outputs
  $$\begin{aligned} ({\dot{b}}, {\dot{b}})(b, b)(a, {\dot{a}})({\dot{a}}, a)(b, {\dot{b}})({\dot{b}}, b)(b, {\dot{b}})(a, a)({\dot{a}}, a)(\#, \#)(\#, \#)\dots \end{aligned}$$
  The next trace, which is the one with $\lnot i$ in front and then  is
  $$\begin{aligned} ({\dot{a}}, {\dot{a}})(b, a)({\dot{b}}, {\dot{b}})(b, b)(a, {\dot{b}})({\dot{a}}, a)(\#, a)(\#, \#)(\#, \#)\dots \end{aligned}$$
  Note that now the symbols are not aligned any more because the first stone did not have an equal length of the upper and lower part. We continue this sequence by removing the next stones:
  $$\begin{aligned}&({\dot{b}}, {\dot{b}})(b, b)(a, {\dot{b}})({\dot{a}}, a)(\#, a)(\#, \#)(\#, \#)\dots \\&({\dot{a}}, {\dot{b}})(\#, a)(\#, a)(\#, \#)(\#, \#)\dots \\&(\#, \#)(\#, \#)\dots \end{aligned}$$
  Now, the formula $\varphi _{ stone \& shift }(\pi ,\pi ')$ encodes that $\pi '$ is $\pi$ with the first stone removed and the rest shifted as illustrated in the example. This can be done with a disjunction over all possible stones. See [18] for more details.
cuts off the irrelevant prefix until $\varphi$ starts. This irrelevant prefix is exactly the part where the $\lnot i$ appear. We are only interested in looking at the  part of the traces because they are not shared by the different *relevant* traces.
- We furthermore assume that only singletons are allowed what can be achieved by a disjunction for each pair of atomic propositions. See [18] as well.

With this construction, we now force by the synthesis algorithm to yield a list of paths which represent the PCP solution step-wise. To get the list of stones used for this PCP solution, we can just examine the first path (with ) and look at the outputs. As we know that the dots above the letters indicate a new stone, we can slice the sequence $i_\alpha$ to $\alpha _{i_1}\dots \alpha _{i_K}$ and same for $i_\beta$. This shows that we can solve PCP if we can solve the synthesis problem for $\forall ^1\exists ^1$ formulas. $\square$

## Bounded realizability

We propose an algorithm to synthesize strategies from specifications given in universal $\text {HyperLTL}$ by searching for finite generators of realizing strategies. We encode this search as a satisfiability problem for a decidable constraint system.

### Transition systems

A *transition system*${\mathcal {S}}$ is a tuple ${\langle S,s_0,\tau ,l \rangle }$ where *S* is a finite set of states, $s_0 \in S$ is the designated initial state, $\tau :S \times 2^{I} \rightarrow S$ is the transition function, and $l :S \rightarrow 2^{O}$ is the state-labeling or output function. We generalize the transition function to sequences over $2^{I}$ by defining $\tau ^* :(2^{I})^* \rightarrow S$ recursively as $\tau ^*(\epsilon ) = s_0$ and $\tau ^*(w_0 \cdots w_{n-1} w_n) = \tau (\tau ^*(w_0 \cdots w_{n-1}), w_n)$ for $w_0 \cdots w_{n-1} w_n \in (2^{I})^+$. A transition system ${\mathcal {S}}$*generates* the strategy *f* if $f(w) = l(\tau ^*(w))$ for every $w \in (2^{I})^*$. A strategy *f* is called *finite-state* if there exists a transition system that generates *f*.

### Overview

We first sketch the synthesis procedure and then proceed with a description of the intermediate steps. Let $\varphi$ be a universal $\text {HyperLTL}$ formula $\forall \pi _1 \cdots \forall \pi _n \mathpunct {.}\psi$. We build the automaton ${\mathcal {A}}_\psi$ whose language is the set of tuples of traces that satisfy $\psi$. We then define the acceptance of a transition system ${\mathcal {S}}$ on ${\mathcal {A}}_\psi$ by means of the self-composition of ${\mathcal {S}}$. Lastly, we encode the existence of a transition system accepted by ${\mathcal {A}}_\psi$ as an SMT constraint system.

#### Example 1

**Fig. 4:**
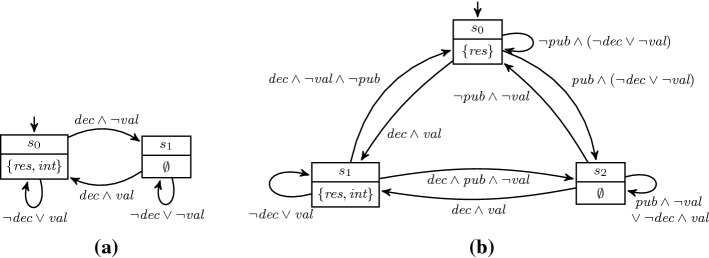
Synthesized Moore transition systems based on the specification given in Example 1

Throughout this section, we will use the following (simplified) running example. Assume we want to synthesize a system that keeps decisions secret until it is allowed to publish. Thus, our system has three input signals *decision*, indicating whether a decision was made, the secret *value*, and a signal to *publish* results. Furthermore, our system has two outputs, an undisclosed output *internal* that stores the value of the last decision, and a public output *result* that indicates the result. No information about decisions should be inferred until publication. To specify the functionality, we propose the $\text {LTL}$ specification3The solution produced by the LTL synthesis tool BoSy [17], shown in Fig. 4a, clearly violates our intention that results should be secret until publish: Whenever a decision is made, the output *result* changes as well.

We formalize the property that no information about the decision can be inferred from *result* until publication as the $\text {HyperLTL}$ formula4It asserts that for every pair of traces, the *result* signals have to be the same until (if ever) there is a *publish* signal on either trace. A solution satisfying both, the functional specification and the hyperproperty, is shown in Fig. 4b. The system switches states whenever there is a decision with a different value than before and only exposes the decision in case there is a prior publish command.

We proceed with introducing the necessary preliminaries for our algorithm.

### Automata

**Fig. 5:**
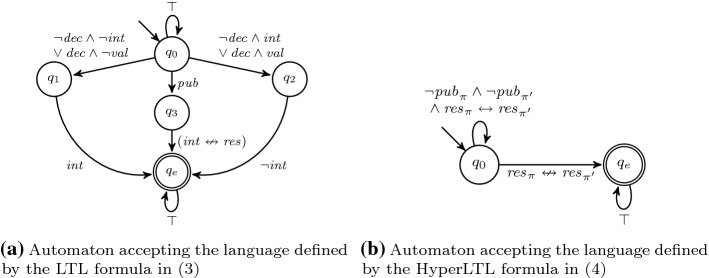
Universal co-Büchi automata recognizing the languages from Example 1

A universal co-Büchi automaton ${\mathcal {A}}$ over finite alphabet $\varSigma$ is a tuple ${\langle Q,q_0,\delta ,F \rangle }$, where *Q* is a finite set of states, $q_0 \in Q$ the designated initial state, $\delta \subseteq Q \times \varSigma \times Q$ is the transition relation, and $F \subseteq Q$ is the set of rejecting states. Given an infinite word $\sigma \in \varSigma ^\omega$, a run of $\sigma$ on ${\mathcal {A}}$ is a finite or infinite path $q_0 q_1 q_2 \dots \in (Q^* \cup Q^\omega )$ where for all $i \ge 0$ it holds that $(q_i,\sigma _i,q_{i+1}) \in \delta$. A run is accepting, if it contains only finitely many rejecting states, i.e., either the run is finite or there exists a $i \ge 0$ such that for all $j \ge i$ it holds that $q_j \notin F$. ${\mathcal {A}}$ accepts a word $\sigma$, if *all* runs of $\sigma$ on ${\mathcal {A}}$ are accepting. The language of ${\mathcal {A}}$, written ${\mathcal {L}}({\mathcal {A}})$, is the set $\{\sigma \in \varSigma ^\omega \mid {\mathcal {A}}\text { accepts } \sigma \}$. We represent automata as directed graphs with vertex set *Q* and a symbolic representation of the transition relation $\delta$ as propositional formulas ${\mathbb {B}}(\varSigma )$. The rejecting states in *F* are marked by double lines. The automata for the $\text {LTL}$ and $\text {HyperLTL}$ specifications from Example 1 are depicted in Fig. 5.

### Run graph

The run graph of a transition system ${\mathcal {S}}= {\langle S,s_0,\tau ,l \rangle }$ on a universal co-Büchi automaton ${\mathcal {A}}= {\langle Q,q_0,\delta ,F \rangle }$ is a directed graph ${\langle V,E \rangle }$ where $V = S \times Q$ is the set of vertices and $E \subseteq V \times V$ is the edge relation with

$$\begin{aligned}&((s, q),(s',q')) \in E \;\text { iff }\;\\&\quad \exists i \in 2^I \mathpunct {.}\exists o \in 2^O \mathpunct {.}(\tau (s,i) = s') \wedge (l(s) = o) \wedge (q,i \cup o,q') \in \delta . \end{aligned}$$

A run graph is accepting if every path (starting at the initial vertex $(s_0,q_0)$) has only finitely many visits of rejecting states. To show acceptance, we annotate every reachable node in the run graph with a natural number *m*, such that any path, starting in the initial state, contains less than *m* visits of rejecting states. Such an annotation exists if, and only if, the run graph is accepting [28].

### Self-composition

The model checking of universal $$\text {HyperLTL}$$ formulas [26] is based on self-composition. Let $$ prj _i$$ be the projection to the *i*-th element of a tuple. Let $$ zip $$ denote the usual function that maps an *n*-tuple of sequences to a single sequence of *n*-tuples, for example, $$ zip ([1, 2, 3], [4, 5, 6]) = [(1, 4), (2, 5), (3, 6)]$$, and let $$ unzip $$ denote its inverse. The transition system $${\mathcal {S}}^n$$ is the *n*-fold self-composition of $${\mathcal {S}}= {\langle S,s_0,\tau ,l \rangle }$$, if $${\mathcal {S}}^n = {\langle S^n, s_0^n, \tau ', l^n \rangle }$$ and for all $$s, s' \in S^n$$, $$\alpha \in (2^{I})^n$$, and $$\beta \in (2^{O})^n$$ we have that $$\tau '(s,\alpha ) = s'$$ and $$l^n(s) = \beta $$ iff for all $$1 \le i \le n$$, it holds that $$\tau ( prj _i(s), prj _i(\alpha )) = prj _i(s')$$ and $$l( prj _i(s)) = prj _i(\beta )$$. If *T* is the set of traces generated by $${\mathcal {S}}$$, then $$\{ zip (t_1,\dots ,t_n) \mid t_1,\dots ,t_n \in T \}$$ is the set of traces generated by $${\mathcal {S}}^n$$.

We construct the universal co-Büchi automaton ${\mathcal {A}}_\psi$ such that the language of ${\mathcal {A}}_\psi$ is the set of words *w* such that $unzip (w) = \varPi$ and $\varPi \vDash _\emptyset \psi$, i.e., the tuples of traces that satisfy $\psi$. We get this automaton by dualizing the non-deterministic Büchi automaton for $\lnot \psi$ [28], i.e., changing the branching from non-deterministic to universal and the acceptance condition from Büchi to co-Büchi. Hence, ${\mathcal {S}}$ satisfies a universal $\text {HyperLTL}$ formula $\varphi = \forall \pi _1 \cdots \forall \pi _n \mathpunct {.}\psi$ if the traces generated by self-composition ${\mathcal {S}}^n$ are a subset of ${\mathcal {L}}({\mathcal {A}}_\psi )$.

#### Lemma 3

A transition system $${\mathcal {S}}$$ satisfies the universal $$\text {HyperLTL}$$ formula $$\varphi =$$$$\forall \pi _1 \cdots \forall \pi _n \mathpunct {.}\psi $$, if, and only if, the run graph of $${\mathcal {S}}^n$$ on $${\mathcal {A}}_\psi $$ is accepting.

### Synthesis

Let ${\mathcal {S}}= {\langle S,s_0,\tau ,l \rangle }$ and ${\mathcal {A}}_\psi = {\langle Q,q_0,\delta ,F \rangle }$. We encode the synthesis problem as an SMT constraint system. Therefore, we use uninterpreted function symbols to encode the transition system and the annotation. For the transition system, those functions are the transition function $\tau : S \times 2^{I} \rightarrow S$ and the labeling function $l: S \rightarrow 2^{O}$. The annotation is split into two parts, a reachability constraint $\lambda ^{\mathbb {B}}: S^n \times Q \rightarrow {\mathbb {B}}$ indicating whether a vertex in the run graph is reachable and a counter $\lambda ^\# : S^n \times Q \rightarrow {\mathbb {N}}$ that maps every reachable vertex to the maximal number of rejecting vertices visited by any path starting in the initial vertex. The resulting constraint asserts that there is a transition system with an accepting run graph.

$$\begin{aligned}&\forall s, s' \in S^n \mathpunct {.}\forall q, q' \in Q \mathpunct {.}\forall i \in (2^{I})^n \mathpunct {.}\\&\left( \lambda ^{\mathbb {B}}(s, q) \wedge \tau '(s, i) = s' \wedge (q, i \cup l(s), q') \in \delta \right) \rightarrow \lambda ^{\mathbb {B}}(s', q') \wedge \lambda ^\#(s', q') \trianglerighteq \lambda ^\#(s, q) \end{aligned}$$

where $\trianglerighteq$ is > if $q' \in F$ and $\ge$ otherwise.

#### Theorem 11

The constraint system is satisfiable with bound *b* if, and only if, there is a transition system ${\mathcal {S}}$ of size *b* that realizes the $\text {HyperLTL}$ formula.

We extract a realizing implementation by asking the satisfiability solver to generate a model for the uninterpreted functions that encode the transition system.

## Bounded unrealizability

So far, we focused on the positive case, providing an algorithm for finding small solutions, if they exist. In this section, we shift to the case of detecting if a universal $\text {HyperLTL}$ formula is unrealizable. We adapt the definition of counterexamples to realizability for $\text {LTL}$ [29] to $\text {HyperLTL}$ in the following. Let $\varphi$ be a universal $\text {HyperLTL}$ formula $\forall \pi _1 \cdots \forall \pi _n \mathpunct {.}\psi$ over inputs *I* and outputs *O*, a *counterexample to realizability* is a set of input traces ${\mathcal {P}}\subseteq (2^I)^\omega$ such that for every strategy $f :(2^{I})^* \rightarrow 2^{O}$ the labeled traces ${\mathcal {P}}^f \subseteq (2^{I \cup O})^\omega$ satisfy $\lnot \varphi = \exists \pi _1 \cdots \exists \pi _n \mathpunct {.}\lnot \psi$.

### Proposition 1

A universal $\text {HyperLTL}$ formula $\varphi = \forall \pi _1 \cdots \forall \pi _n \mathpunct {.}\psi$ is unrealizable if, and only if, there is a counterexample ${\mathcal {P}}$ to realizability.

### Proof

Let ${\mathcal {P}}$ be a counterexample to realizability. Assume for contradiction that $\varphi$ is realizable by a strategy *f*. By definition of ${\mathcal {P}}$, we know that ${\mathcal {P}}^f \vDash \exists \pi _1 \cdots \exists \pi _n \mathpunct {.}\lnot \psi$. This means that there exists an assignment $\varPi _{\mathcal {P}}:{\mathcal {V}}\rightarrow {\mathcal {P}}^f$ with $\varPi _{\mathcal {P}}\vDash _{{\mathcal {P}}^f} \lnot \psi$, which is equivalent to $\varPi _{\mathcal {P}}\nvDash _{{\mathcal {P}}^f} \psi$. Therefore, not all assignments $\varPi : {\mathcal {V}}\rightarrow {\mathcal {P}}^f$ satisfy $\varPi \vDash _{{\mathcal {P}}^f} \psi$, which implies that ${\mathcal {P}}^f \nvDash \forall \pi _1 \cdots \forall \pi _n \mathpunct {.}\psi = \varphi$. Hence, $f \nvDash \varphi$, which concludes the contradiction.

Let $\varphi$ be unrealizable. We show that the set ${\mathcal {P}}= (2^I)^\omega$ is a counterexample to realizability. Let $f :(2^{I})^* \rightarrow 2^{O}$ be an arbitrary strategy, and let ${\mathcal {P}}^f$ be the corresponding set of labeled traces. From the unrealizability of $\varphi$, we now that $f \nvDash \forall \pi _1 \cdots \forall \pi _n \mathpunct {.}\psi$. Thus, there exists a trace assignment $\varPi _{\mathcal {P}}:{\mathcal {V}}\rightarrow {\mathcal {P}}^f$ with $\varPi _{\mathcal {P}}\vDash _{{\mathcal {P}}^f} \lnot \psi$, which is equivalent to ${\mathcal {P}}\vDash \exists \pi _1 \cdots \exists \pi _n \mathpunct {.}\lnot \psi$. $\square$

Despite being independent of strategy trees, there are in many cases finite representations of ${\mathcal {P}}$. Consider, for example, the unrealizable specification , where the set ${\mathcal {P}}_1 = \{ \emptyset ^\omega , \{i\}^\omega \}$ is a counterexample to realizability. As a second example, consider  with conflicting requirements on *o*. ${\mathcal {P}}_1$ is a counterexample to realizability for $\varphi _2$ as well: By choosing a different valuation of *i* in the first step of ${\mathcal {P}}_1$, the system is forced to either react with different valuations of *o* (violating the first conjunct), or not correctly repeating the initial value of *i* (violating the second conjunct).

**Fig. 6:**
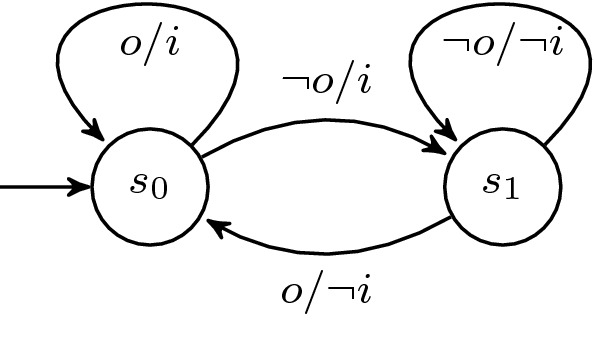
Counterexample strategy for (5)

There are, however, already linear specifications where the set of counterexample traces is not finite and depends on the strategy tree [30]. For example, the specification5is unrealizable as the system cannot predict future values of the environment. There is no finite set of traces witnessing this: For every finite set of traces, there is a strategy tree such that  holds on every such trace. On the other hand, there is a simple *counterexample strategy*, that is a strategy that observes output sequences and produces inputs, depicted in Fig. 6. In this example, the counterexample strategy inverts the outputs given by the system, thus it is guaranteed that  for every system strategy.

We combine those two approaches, selecting counterexample traces and using strategic behavior. A *k*-counterexample strategy for $\forall ^n\text {HyperLTL}$ observes *k* output sequences and produces *k* inputs, where *k* is a new parameter. We require that *k* is at least the number of universal quantifiers *n*. The counterexample strategy is winning if (1) either the traces given by the system player do not correspond to a strategy, or (2) the body of the $\text {HyperLTL}$ formula is violated for any *n* subset of *k* traces. Regarding property (1), consider the two traces where the system player produces different outputs initially. Clearly, those two traces cannot be generated by any system strategy since the initial state (root labeling) is fixed.

We reduce the search for a *k*-counterexample strategy to LTL synthesis. For every atomic proposition $a \in \text {AP}$, we produce *k* copies $a^1,\ldots ,a^k$. We use the same notation for sets of atomic propositions, e.g., $I^j = \{i^j \mid i \in I\}$ for $1 \le j \le k$. The search for a *k*-counterexample strategy can be reduced to LTL synthesis using *k*-tuple input propositions $O^k$, *k*-tuple output propositions $I^k$, and the formula

$$\begin{aligned} { strategic }(I^k, O^k) \rightarrow \bigvee _{P \subseteq \{1,\dots ,k\} \text { with } {|P|} = n} \lnot \psi [P] , \end{aligned}$$

where $\psi [P]$ denotes the replacement of $a_{\pi _i}$ by the $P_i\hbox {th}$ position of the combined input/output *k*-tuple. The formula ${ strategic }(I^k, O^k)$ enforces that the behavior of the system player is strategic and is defined asThis is an instance of the formula (sym) given in Sect. 3.

### Theorem 12

A universal $\text {HyperLTL}$ formula $\varphi = \forall \pi _1 \cdots \forall \pi _n \mathpunct {.}\psi$ is unrealizable if there is a *k*-counterexample strategy for some $k \ge n$.

### Proof

Fix $\varphi$ and let $f_{ cex } :(2^{O^k})^+ \rightarrow 2^{I^k}$ be a *k*-counterexample strategy. Assume for contradiction that $f :(2^{I})^* \rightarrow 2^{O}$ is a strategy realizing $\varphi$. Let $f^k :(2^{I^k})^* \rightarrow 2^{O^k}$ be the strategy that represents the *k*-fold self-composition of *f* (adapting atomic propositions as described earlier). By combining *f* and $f_{ cex }$, we get an infinite sequence $t \in (2^{I^k \cup O^k})^\omega$: $t_0 = f(\epsilon ) \cup f_{ cex }(f(\epsilon )), t_1 = f(f_{ cex }(f(\epsilon ))) \cup f_{ cex }(f(f_{ cex }(f(\epsilon )))), \ldots$ This sequence represents a *k*-tuple ${ cex }_k = (I \cup O)^k$. As $f^k$ satisfies ${ strategic }(I^k, O^k)$, there is a *n*-tuple ${ cex }_n$ build from elements of ${ cex }_k$ such that for the corresponding trace assignment $\varPi$ it holds that $\varPi \vDash \lnot \psi$. This contradicts our assumption that *f* is a realizing strategy. $\square$

## Evaluation

We implemented a prototype synthesis tool, called $\text {BoSyHyper}$,^1^ for universal $\text {HyperLTL}$ based on the bounded synthesis algorithm described in Sect. 5. Furthermore, we implemented the search for counterexamples proposed in Sect. 6. Thus, $\text {BoSyHyper}$ is able to characterize realizability and unrealizability of universal $\text {HyperLTL}$ formulas.

We base our implementation on the LTL synthesis tool BoSy [17]. For efficiency, we split the specifications into two parts, a part containing the linear (LTL) specification, and a part containing the hyperproperty given as $\text {HyperLTL}$ formula. Consequently, we build two constraint systems, one using the standard bounded synthesis approach [28] and one using the approach described in Sect. 5. Before solving, those constraints are combined into a single SMT query. This results in a much more concise constraint system compared to the one where the complete specification is interpreted as a $\text {HyperLTL}$ formula. For solving the SMT queries, we use the Z3 solver [12]. We continue by describing the benchmarks used in our experiments.

### Symmetric mutual exclusion

**Fig. 7:**
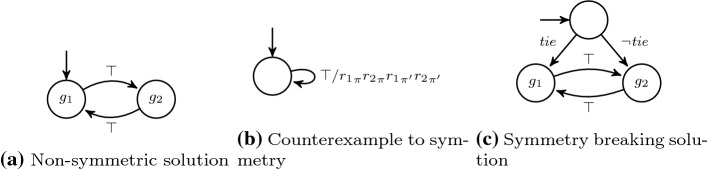
Synthesized solution of the mutual exclusion protocols

Our first example demonstrates the ability to specify symmetry in $\text {HyperLTL}$ for a simple mutual exclusion protocol. Let $r_1$ and $r_2$ be input signals representing mutually exclusive *requests* to a critical section and $g_1/g_2$ the respective grants to enter the section. Every request should be answered eventually  for $i \in \{1,2\}$, but not at the same time . The minimal LTL solution is depicted in Fig. 7a. It is well known that no mutex protocol can ensure perfect symmetry [37], thus when adding the symmetry constraint specified by the $\text {HyperLTL}$ formula  the formula becomes unrealizable. Our tool produces the counterexample shown in Fig. 7b. By adding another input signal *tie*, that breaks the symmetry in case of simultaneous requests and modifying the symmetry constraint  we obtain the solution depicted in Fig. 7c. We further evaluated the same properties on a version that forbids spurious grants, which are reported in Table 2 with prefix *full*.

### Distributed and fault-tolerant systems

**Fig. 8:**
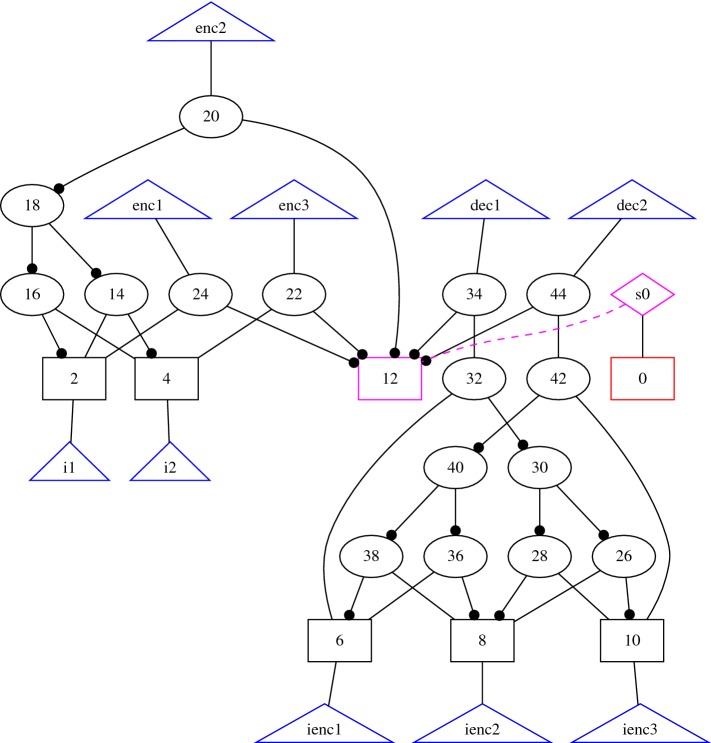
Representation of the solution for an encoder with 2 input bits and 3 encoded bits as And-Inverter-Graph. The solution is produced by BoSyHyper where the specification is given as a single HyperLTL formula specifying both, the encoder and the decoder, as well as the distributivity constraints. Note that although BoSyHyper produces a global implementation, the implementation is actually distributed as decoder and encoder do not share gates

In Sect. 4 we presented a reduction of arbitrary distributed architectures to $\text {HyperLTL}$. As an example for our evaluation, consider a setting with two processes, one for *encoding* input signals and one for *decoding*. Both processes can be synthesized simultaneously using a single $\text {HyperLTL}$ specification. The (linear) correctness condition states that the decoded signal is always equal to the inputs given to the encoder. Furthermore, the encoder and decoder should solely depend on the inputs and the encoded signal, respectively. Additionally, we can specify desired properties about the encoding like fault-tolerance [30] or Hamming distance of code words [26]. An example solution for 2 input bits and 3 encoded bits is shown in Fig. 8. For the encoding, we required that for every change in the input, two encoding bits change. The synthesized solution uses a parity bit as the third encoded bit and the encoding and decoding parts are strictly independent. The results are reported in Table 2 where *i*-*j*-*x* means *i* input bits, *j* encoded bits, and *x* represents the property. The property is either tolerance against a single Byzantine signal failure or a guaranteed Hamming distance of code words.

**Table 2:** Results of BoSyHyper on the benchmarks sets described in Sect. 7

Benchmark	Instance	Result	States		Time [s]	
			Moore	Mealy	Moore	Mealy
Symmetric mutex	Non-sym	Realizable	2	2	1.4	1.3
	Sym	Unrealizable ($k=2$)	1	1	1.9	2.0
	Tie	Realizable	3	3	1.7	1.6
	Full-non-sym	Realizable	4	4	1.4	1.4
	Full-sym	Unrealizable ($k=2$)	1	1	4.3	6.2
	Full-tie	Realizable	9	5	1 802.7	5.2
Encoder/decoder	1-2-Hamming-2	Realizable	4	1	1.6	1.3
	1-2-Fault-tolerant	Unrealizable ($k=2$)	1	–	54.9	–
	1-3-Fault-tolerant	Realizable	4	1	151.7	1.7
	2-2-Hamming-2	Unrealizable $(k=3)$	–	1	–	10.6
	2-3-Hamming-2	Realizable	16	1	$> 1$ h	1.5
	2-3-Hamming-3	Unrealizable $(k=3)$	–	1	–	126.7
CAP Theorem	cap-2-linear	Realizable	8	1	7.0	1.3
	cap-2	Unrealizable $(k=2)$	1	–	1 823.9	–
	ca-2	Realizable	–	1	–	4.4
	ca-3	Realizable	–	1	–	15.0
	cp-2	Realizable	1	1	1.8	1.6
	cp-3	Realizable	1	1	3.2	10.6
	ap-2	Realizable	–	1	–	2.0
	ap-3	Realizable	–	1	–	43.4
Bus protocol	NI1	Unrealizable $(k=2)$	1	1	75.2	69.6
	NI2	Realizable	8	8	24.1	33.9
Dining cryptographers	Secrecy	Realizable	–	1	–	82.4

They ran on a machine with a dual-core Core i7, 3.3 GHz, and 16 GB memory

### CAP Theorem

The CAP Theorem due to Brewer [5] states that it is impossible to design a distributed system that provides Consistency, Availability, and Partition tolerance (CAP) simultaneously. This example has been considered before [30] to evaluate a technique that could automatically detect unrealizability. However, when we drop either Consistency, Availability, or Partition tolerance, the corresponding instances (AP, CP, and CA) become realizable, which the previous work was not able to prove. We show that our implementation can show both, unrealizability of CAP and realizability of AP, CP, and CA. In contrast to the previous encoding [30] we are not limited to acyclic architectures.

### Long-term information flow

Previous work on model-checking hyperproperties [26] found that an implementation for the commonly used *I2C* bus protocol could remember input values ad infinitum. For example, it could not be verified that information given to the implementation eventually leaves it, i.e., is forgotten. This is especially unfortunate in high security contexts. We consider a simple bus protocol which is inspired by the widely used *I2C* protocol. Our example protocol has the inputs *send* for initiating a transmission, *in* for the value that should be transferred, and an *ack*nowledgment bit indicating successful transmission. The bus master waits in an *idle* state until a *send* is received. Afterwards, it transmits a header sequence, followed by the value of *in*, waits for an acknowledgement and then indicates *success* or *failure* to the sender before returning to the idle state. We specify the property that the *in*put has no influence on the *data* that is send, which is obviously violated (instance NI1). As a second property, we check that this information leak cannot happen arbitrary long (NI2) for which there is a realizing implementation.

### Dining cryptographers

Recap the dining cryptographers problem introduced earlier. This benchmark is interesting as it contains two types of hyperproperties. First, there is information flow between the three cryptographers, where some secrets ($s_{ab}, s_{ac}, s_{bc}$) are shared between pairs of cryptographers. In the formalization, we have 4 entities: three processes describing the 3 cryptographers ($\textit{out}_i$) and one process computing the result ($p_g$), i.e., whether the group has paid or not, from $\textit{out}_i$. Second, the final result should only disclose whether one of the cryptographers has paid or the NSA. This can be formalized as a indistinguishability property between different executions. For example, when we compare the two traces $\pi$ and $\pi '$ where $C_a$ has paid on $\pi$ and $C_b$ has paid on $\pi '$, then the outputs of both have to be the same, if their common secret $s_{ab}$ is different on those two traces (while all other secrets $s_{ac}$ and $s_{bc}$ are the same). This ensures that from an outside observer, a flipped output can be either result of a different shared secret or due to the announcement. Lastly, the linear specification asserts that $p_g \leftrightarrow \lnot p_\textit{NSA}$.

**Fig. 9:**
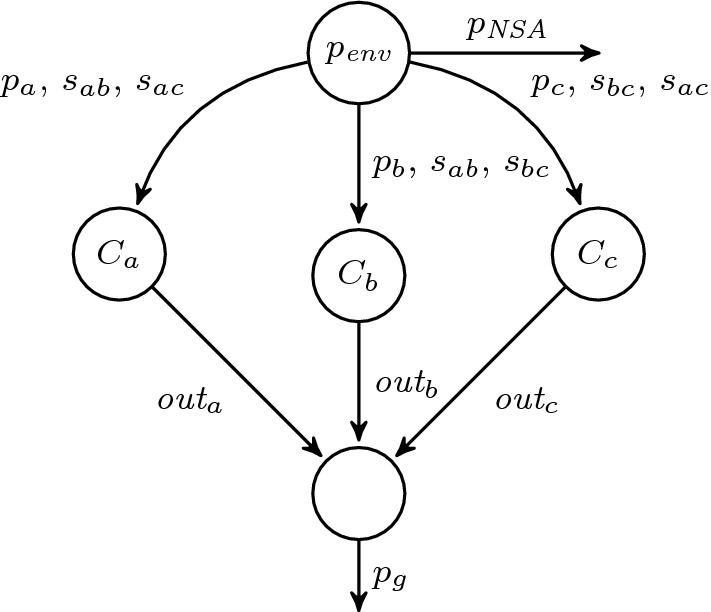
Architecture for the dining cryptographers

This question can be encoded as a synthesis problem for $\text {HyperLTL}$. What makes this example particularly interesting is the combination of multiple *information*-*flow* requirements:

1. The setting is *distributed*, we have four entities: The three cryptographers ($C_a$, $C_b,$ and $C_c$), where each cryptographer shares a secret bit with each other (denoted $s_{ab}$ for the shared secret of $C_a$ and $C_b$). The fourth entity is the process that receives the output from the cryptographers ($\textit{out}_a$, $\textit{out}_b$, and $\textit{out}_c$) and computes the result whether one of them has paid the bill (output $p_g$). Figure 9 gives a visual representation of this distributed architecture.
2. The secrecy requirement is formalized as requiring *indistinguishability* between different executions for an outside party (observing $\textit{out}_a$, $\textit{out}_b$, and $\textit{out}_c$). For example, when we compare the two execution traces $\pi$ and $\pi '$ where $C_a$ has paid on $\pi$ and $C_b$ has paid on $\pi '$. Then the outputs of both have to be the same, if their common secret $s_{ab}$ is different on those two traces (while all other secrets $s_{ac}$ and $s_{bc}$ are the same). This ensures that from an outside observer, a flipped output can be either result of a different shared secret or due to the announcement.

We now formalize this as a $\text {HyperLTL}$ synthesis problem. The set of atomic propositions is partitioned into environment outputs $I = \{p_\textit{NSA}, p_a, p_b, p_c, s_{ab}, s_{ac}, s_{bc}\}$ and system outputs $O = \{\textit{out}_a, \textit{out}_b, \textit{out}_c, p_g\}$. The functional ($\text {LTL}$) requirements are simple, assuming that exactly one of $p_\textit{NSA}$, $p_a$, $p_b$, and $p_c$ is true, the output of $p_g$ is the negation of $p_\textit{NSA}$. As an $\text {LTL}$ formulaThe distributed architecture is encoded as a conjunction of $\text {HyperLTL}$ formulas ensuring independence of non-observable inputs, i.e.,

$$\begin{aligned} \forall \pi \mathpunct {.}\forall \pi '\mathpunct {.}&D^{\pi ,\pi '}_{\{p_a,s_{ab},s_{ac}\} \mapsto \{\textit{out}_a\}} \wedge D^{\pi ,\pi '}_{\{p_b,s_{ab},s_{bc}\} \mapsto \{\textit{out}_b\}}\\ \wedge&D^{\pi ,\pi '}_{\{p_c,s_{ac},s_{bc}\} \mapsto \{\textit{out}_c\}} \wedge D^{\pi ,\pi '}_{\{\textit{out}_a,\textit{out}_b,\textit{out}_c\} \mapsto \{p_g\}} \end{aligned}$$

Lastly, the indistinguishability (exemplified for $C_a$ and $C_b$) is formalized asfor every pair of cryptographers.

Neither $\text {LTL}$ synthesis nor its distributed variant can express the combination of those requirements. Our $\text {HyperLTL}$ synthesis tool $\text {BoSyHyper}$ [20] is able to find a solution to this problem. A closer look in the implementation reveals, that the tool has synthesized the XOR scheme presented in the original solution [6].

### Results

Table 2 reports on the results of the benchmarks. We distinguish between state-labeled (*Moore*) and transition-labeled (*Mealy*) transition systems. Note that the counterexample strategies use the opposite transition system, i.e., a Mealy system strategy corresponds to a state-labeled (Moore) environment strategy. Typically, Mealy strategies are more compact, i.e., need smaller transition systems and this is confirmed by our experiments. BoSyHyper is able to solve most of the examples, providing realizing implementations or counterexamples. Regrading the unrealizable benchmarks we observe that usually two simultaneously generated paths ($k=2$) are enough with the exception of the encoder example. Overall the results are encouraging showing that we can solve a variety of instances with non-trivial information flow.

## Conclusion

In this paper, we have studied the reactive realizability problem for specifications given in the temporal logic $\text {HyperLTL}$. We showed that this problem subsumes various extensions of the LTL realizability problem: synthesis under incomplete information, distributed synthesis, symmetric synthesis, and fault-tolerant synthesis can all be encoded in the synthesis problem of HyperLTL. We gave a complete characterization of the decidable fragments based on the quantifier prefix and, additionally, identified a decidable fragment in the, in general undecidable, universal fragment of $\text {HyperLTL}$. Furthermore, we presented two algorithms to detect realizable and unrealizable $\text {HyperLTL}$ specifications, one based on bounding the system implementation and one based on bounding the number of counterexample traces. Our prototype implementation shows that our approach is able to synthesize systems with complex information-flow properties.

## Footnotes

1 $\text {BoSyHyper}$ is available at https://www.react.uni-saarland.de/tools/bosy/.
